# Social exclusion evokes different psychophysiological responses in individuals high on the psychopathy facets fearless dominance and self-centered impulsivity

**DOI:** 10.3389/fpsyt.2023.1197595

**Published:** 2024-01-11

**Authors:** Natalia A. Seeger, Nathalie Brackmann, Claus Lamm, Kristina Hennig-Fast, Daniela M. Pfabigan

**Affiliations:** ^1^Social Cognitive and Affective Neuroscience Unit, Department of Cognition, Emotion, and Methods in Psychology, Faculty of Psychology, University of Vienna, Vienna, Austria; ^2^Department of Forensic Psychiatry, University Hospital of Psychiatry Zurich, Zurich, Switzerland; ^3^Department of Psychiatry and Psychotherapy, University of Bielefeld, Bielefeld, Germany; ^4^Department of Biological and Medical Psychology, Faculty of Psychology, University of Bergen, Bergen, Norway

**Keywords:** primary psychopathy, secondary psychopathy, EDA, emotion regulation, ostracism

## Abstract

Individuals with psychopathic personality traits are generally thought to have difficulties in processing and experiencing emotions. These difficulties could also translate to emotionally charged social situations such as social exclusion. Being socially excluded is often experienced as stressful and unpleasant, potentially even leading to selfish or aggressive behavior–both of which are linked to certain aspects of psychopathy. The current study investigated self-report and physiological responses to social exclusion in the cyberball paradigm in a carefully selected community sample of individuals either scoring high on primary (*N* = 24) or secondary psychopathy traits (*N* = 17). Across the sample, the cyberball paradigm decreased experiences of joy and approach motivation, increased subjective anger reports, and induced changes in heart rate. In contrast, individuals scoring high on secondary psychopathy traits (Self-Centered Impulsivity group) displayed stronger physiological reactivity during a habituation phase of prolonged social exclusion than individuals scoring high on primary psychopathy traits (Fearless Dominance group), indexed by changes in skin conductance level. Moreover, a potential mismatch between self-reported and physiological arousal seemed to be only observable in individuals with high secondary psychopathy traits. Overall, the current results suggest diverging patterns of emotional processing and regulation in a social exclusion situation when comparing well-functioning individuals with varying psychopathy traits. It seemed as if individuals high on primary psychopathy traits were insensitive to contextual social cues, while individuals high on secondary psychopathy traits were more affected by the potentially threatening social situation. Cautiously transferring the current findings to forensic samples, they support the idea of moving from a behavioral understanding of the psychopathy construct to a more clinical picture with distinct cognitive and emotional processing patterns in individuals high on either primary or secondary psychopathy traits.

## 1 Introduction

Previous research suggests that individuals have an evolutionary grounded *need to belong* to social groups or persons and hence seek stable, frequent, and positive social interactions and relationships (1). Thus, the question arises of what happens when one’s need to belong is violated or threatened. In experimental situations of social exclusion, individuals immediately react with enhanced frustration [e.g., (2)], distress (3), physiological arousal [e.g., (4)], as well as self-reported [e.g., (5)] and behavioral [e.g., (6)] aggression. As a physiological reaction to social exclusion, among others, individuals show increases in tension and diastolic and systolic blood pressure (7).

Being socially excluded also has an influence on prosocial or antisocial behavior to the point that “when the benefits of prosocial behavior are not linked to possible acceptance, socially excluded people behave selfishly” [(8), p. 986]. Hence, whether someone *feels* socially excluded plays a pivotal role in their behavior. In general, emotional processing and responding play a central role in human social interactions (9–11). This notion is of particular importance in individuals with problems in emotion processing. Among others, difficulties in emotional processing are linked to the construct of psychopathy (12–15), which in turn is directly linked to antisocial behavior (16, 17).

Previous studies have investigated how individuals with psychopathic traits process emotional information [e.g., (18–20)], their subjective emotional experience [e.g., (21)], and their physiological reactions to emotional stimuli (16, 22). The construct of psychopathy is based on different theoretical concepts (23, 24). The terminology describing different psychopathic subtypes varies regarding the underlying theoretical model, the measure used to capture psychopathic traits,^1^ and the investigated sample (e.g., forensic sample, normal population, youth and adolescents). To account for these different theoretical approaches, the terms *primary psychopathy* and *secondary psychopathy* are used to describe general results regarding the two main subtypes of psychopathic individuals. It has to be noted though that the characteristics of primary and secondary psychopathy are not always fully represented in the respective factors measured by different psychopathy scales. Primary psychopathy outlines the affective-interpersonal facet of psychopathy and is associated with traits such as manipulative behavior, lack of empathy, guilt, and fearlessness. Secondary psychopathy outlines the behavioral-lifestyle facets of the construct and is associated with emotional difficulties that are reflected in enhanced impulsivity, frustration intolerance, aggressive behavior, and a lack of responsibility (27–34). The current study approximates primary psychopathy traits via the Fearless Dominance Scale and secondary psychopathy traits via the Self-Centered Impulsivity Scale of the Psychopathic Personality Inventory-Revised [PPI-R; (35, 36)]. Differentiating different subtypes or trait components of psychopathy is crucial because they often show contrasting or even opposing associations with experimental measures such as self-report, task performance, or physiological correlates (37).

Depending on the subtype, psychopathic individuals seem to adapt differently to social situations. In a community sample, individuals with primary psychopathy traits seem to not suffer from social functioning difficulties due to their personality traits, whereas individuals with secondary psychopathic traits exhibit a lack of success in exactly this area (12). Moreover, conflict-laden social situations (such as interpersonal conflict) can cause diverging behavioral and physiological reactions in individuals scoring high on either primary or secondary psychopathy traits. While individuals with primary psychopathy traits seem to inhibit anger in social conflicts, individuals with secondary psychopathy traits seem to show increased risk for anger (38). Furthermore, individuals with high primary psychopathy traits are considered to show diminished startle reflex responses [e.g., (14)], as well as lower electrodermal activity (EDA) to aversive stimuli [e.g., (39)]. In contrast, high secondary psychopathic traits have been associated with enhanced startle reflex responses toward negatively valenced stimuli [e.g., (15)], but also with reduced electrodermal activity in response to all stimuli irrespective of their valence [e.g., (39)]. However, a recent meta-analysis reported no compelling evidence for clear directional effects when comparing individuals with primary and secondary psychopathy traits in regards to psychophysiological responses (40).

When it comes to emotion regulation, recent research reported global difficulties of individuals with psychopathy traits to adapt their perception or behavior in emotional situations accordingly, especially when their attention had already been directed toward an emotional stimulus (41, 42). Individuals with primary psychopathy traits commonly showed less interest toward emotional information and therefore paid less attention to it (41, 43). In contrast, individuals with secondary psychopathy traits were considered to show difficulties in inhibitory processes relevant in the context of emotions, impacting their emotion regulation and emotion expression (44, 45). Casey et al. (22) investigated emotional experiencing and suppression in individuals high on psychopathic traits and reported a reduced ability to experience negative emotions in individuals with primary psychopathy traits only. According to self-reported emotional coping processes, individuals with primary psychopathy traits tended to experience predominantly positively-valenced states such as pride, strength, enthusiasm etc., whereas individuals with secondary psychopathy traits seemed to experience predominantly negatively-valenced states such as shame, guilt, hostility etc. (46, 47). Overall, while the available evidence paints a complex and in part inconsistent picture, primary psychopathy traits seem to be associated with a reduced ability to experience negative affective states and emotions (22), but also with an enhanced experience of positive affective states and emotions (46, 48), while secondary psychopathy traits are rather associated with negative affective states and emotions (46, 47).

Considering the strong and diverging emotional difficulties that are attributed to both primary and secondary psychopathy traits, and the key role these abilities play in interpersonal and social contexts (49–52), there is still a lack of understanding concerning how individuals with diverging psychopathy traits respond to social frustration and ostracism.^2^ This knowledge gap is of particular importance because psychopathic individuals–in particular when it comes to forensic samples–are associated with high rates of criminal recidivism, which poses considerable challenges regarding their long-term treatment and rehabilitation (54, 55). Even though the prevalence of psychopathic traits in forensic samples (from 3% up to 73%; see (56)) seems to be higher than in community samples [between 0.6% (57); and 4.5% (58)], high scoring non-criminal psychopathic individuals appear not to differ significantly from criminal psychopathic individuals in terms of psychopathic traits *per se*, but only in their degree of expression (59).

In particular individuals with secondary psychopathy traits seem to have difficulties in social interactions and might more often fall victim to social exclusion (60). At the same time, exactly this group of individuals might be more prone to become delinquent and end up in prison, which could be the result of repeated experiences of social exclusion. However, it is still not known whether individuals with primary and secondary psychopathy traits react differently to social exclusion. The current study aims to fill the prevailing knowledge gap by investigating how individuals scoring high on one of the psychopathy subtypes react to social exclusion in an established experimental paradigm, the cyberball task (5). The cyberball is a short experimental ball tossing task that confronts participants with situations and feelings of belonging to a group (while being included and repeatedly receiving the ball) and of social exclusion (while being excluded and active participation in the game is denied). Self-report and psychophysiological responses (electrodermal and cardiac activity) to such a potentially stressing and frustrating event were assessed in a carefully selected male community sample with individuals scoring either high on primary or secondary psychopathy traits. The assessment of peripheral psychophysiological responses was conducted to provide a more objective measurement of arousal and emotional reactions in response to social rejection. A previous study investigated neural processing during social exclusion situations in adolescents and young adults with varying global psychopathy sum scores (61). The authors observed diminished cognitive-affective processing during inclusion trials, but enhanced processing during ambiguous exclusion trials in individuals scoring high on overall psychopathy. Such a differentiation between inclusion and exclusion might also be reflected in peripheral responses assessed in the current study.

Based on previous findings, we assumed a reduced experience of self-reported negative emotional states in individuals with primary psychopathy traits, while individuals with secondary psychopathy traits were assumed to experience an increase in negative emotional states in response to social exclusion [e.g., (15, 62)]. Furthermore, we assumed that individuals with primary psychopathy traits might show generally stronger positive affective states compared to individuals with secondary psychopathy traits [e.g., (46–48)].

Based on more inconsistent previous findings and a recent meta-analysis suggesting high task- and measure-dependency of psychophysiological responses (63), we had no directional hypotheses of how individuals high on primary or secondary psychopathy traits would differ in electrodermal and cardiac activity in response to social exclusion. In addition, we explored whether self-report and psychophysiological responses to social exclusion were associated with each other.

## 2 Materials and methods

### 2.1 Participants

In total, 1,612 volunteers (of which 445 men) completed an online screening. Approximately 15% of the online sample had to be excluded due to exceeding the cut-off score of the PPI-R inconsistent responding scales (64). Since the current study focused on a male-only sample, 372 men were available because of valid questionnaire scores. Within this male sample, those scoring high (similar to the top 25% of a German speaking reference population) on either the Fearless Dominance (*n* = 24) or the Self-Centered Impulsivity (*n* = 17) subscale of the Psychopathic Personality Inventory–Revised [PPI-R; (35)] were selected and invited to the laboratory. Further, we also invited individuals with very low psychopathy traits, represented by low scores (similar to the bottom 25% of a German speaking reference population) on both subscales to the laboratory with the aim to collect a control group. However, only 13 individuals fulfilling these strict criteria could be recruited for the experiment. Furthermore, it can be questioned, if participants of this low-trait group do represent a valid control group, or rather another extreme group characterized by, e.g., emotional sensitivity, high fearfulness and planfulness (as opposites to high manifestations in the single PPI-R scales), more associated with neuroticism. We therefore decided to focus on the comparison between the two high-scoring psychopathy groups in the current manuscript only. Comparisons of the two psychopathy groups to the “low-trait group” are reported in Supplementary materials. Groups were selected based on a validated general reference population of the PPI-R (data and cut-off scores based on a German version of the Psychopathic Personality Inventory–Revised (PPI-R), provided by H. Eisenbarth, personal communication, see Table 1). The final sample size was based on *a priori* considerations of prevalence rates of psychopathic traits in the general population [e.g., (57, 58)]. Statistical power was enhanced by using an extreme-group design and choosing the top 25% of PPI-R Fearless Dominance scores (while controlling the second factor, Self-Centered Impulsivity, for scores around the bottom 25%) for the group high on primary psychopathy traits (Fearless Dominance: *M* = 134.46, SD = 11.88; Self-Centered Impulsivity: *M* = 119.33, SD = 8.92), and the top 25% of PPI-R Self-Centered Impulsivity scores (while controlling the second factor, Fearless Dominance, for scores around the bottom 25%) for the group high on secondary psychopathy traits (Self-Centered Impulsivity: *M* = 157.18, SD = 11.32; Fearless Dominance: *M* = 98.18, SD = 9.73) (65, 66).

**Table 1:** PPI-R cut-off scores of the psychopathy factors for group selection based on a validated general German speaking reference population (provided by H. Eisenbarth, personal communication).

	<25%	50%	>75%
Fearless dominance	101	112	121
Self-centered impulsivity	121	132	145

The study was approved by the University of Vienna ethics committee (nr. 00108, 2015-01-22) and was conducted in accordance with the most recent revision of the Declaration of (67). All participants gave written consent before starting the test session in the laboratory and were financially reimbursed for their participation.

### 2.2 Instruments (self-report)

The Psychopathic Personality Inventory–Revised [PPI-R; (35)] is a 154 item self-report questionnaire, suitable for the assessment of psychopathic traits in community populations (for a meta-analysis on factor structure in community samples see (26)). The eight factors of the PPI-R can be summed up to a general psychopathy score as well as to two higher-order subscales or psychopathy factors, namely Fearless Dominance (FD) consisting of *Social Influence*, *Fearlessness*, and *Stress Immunity*, and Self-Centered Impulsivity (SCI) consisting of *Carefree Nonplanfulness*, *Rebellious Nonconformity*, *Machiavellian Egocentricity*, and *Blame Externalization*. The factor *Coldheartedness* is not assigned to any higher-order factor. Furthermore, the PPI-R provides four scales indicating a systematic or manipulative responding style: two *inconsistent responding* scales (IR15 and IR40), a *virtuous responding* scale (VR), and a *deviant responding* scale [DR; see, e.g., (64, 68)].

We assessed several psychological variables known to be associated with Fearless Dominance and Self-Centered Impulsivity (69, 70) to account for their potential influence on the results: *depression* [Beck Depression Inventory-II; BDI-II; (71)], *social anxiety* [Social-Interaction Anxiety Scale; SIAS; (72)], *impulsivity* [Barratt Impulsiveness Scale II; BIS-II; (73)], and *anger disposition* [State–Trait Anger Expression Inventory; STAXI; (74)].

While at the laboratory, participants completed several subjective state assessments before and after the cyberball paradigm. A non-verbal version of the Self-Assessment Manikin [SAM; (75)] was administered to assess *arousal*, *dominance*, and *joy* on a 9-point-Likert-scale, as well as the Positive and Negative Affect Scale [PANAS; (76)] to assess changes in *positive affect*, *negative affect* and *affective polarity* in response to social exclusion. Affective polarity constitutes a third factor of the traditional PANAS scale where 10 items each load on Positive and Negative Affect. The factor Affective Polarity regroups 10 items of the scale and describes a general tendency to either react with higher approach to a certain stimulus or situation or with higher withdrawal (77). Therefore, higher affective polarity scores can be interpreted as higher approach or lower withdrawal orientation, whereas lower scores can be interpreted as lower approach or higher withdrawal orientation. In addition, the state *anger* scale of the State–Trait Anger Expression Inventory was administered [STAXI; (74)] to assess changes in perceived anger.

### 2.3 Task and procedure

After providing written consent, participants were informed that they were going to participate in an experiment together with several other individuals, who were brought to other laboratory rooms though. These other participants were assigned to groups A and B, while the participant was assigned to group C. The other players were only part of the cover story though. To enhance credibility of the group assignment, photographs of each player were taken which were supposedly uploaded into the experimental software. The photographs of the other two players were always the same. After the experimental part of the study, participants had the chance to ask questions about the purpose of the study. No standardized debriefing took place.^3^

Before starting the experimental paradigm, participants were prepared for the psychophysiological measurements and a 1-min baseline recording at rest (resting phase) of electrodermal activity (EDA) and cardiac activity via an electrocardiogram (ECG) was conducted. Furthermore, participants filled in state self-report questionnaires before and after the experimental paradigm. EDA and ECG were recorded throughout the whole experimental task.

**Figure 1:**
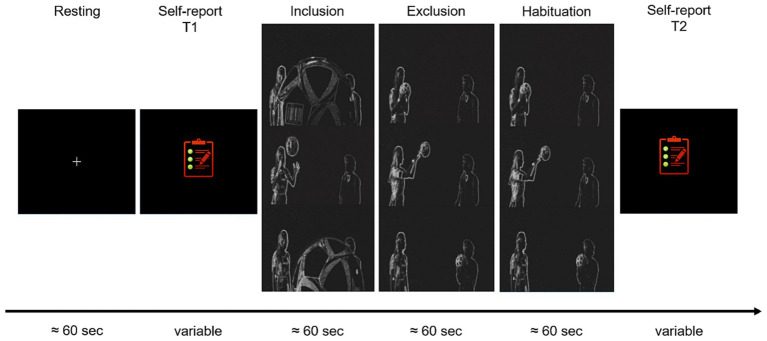
Graphical overview of the experimental timeline of the cyberball paradigm, adapted version by Novembre et al. (79). Electrodermal and cardiac activity were assessed concurrently.

Social exclusion was induced using an adapted version of Williams (78) cyberball paradigm [see (79) for the adapted version], introduced to the participants as a virtual ball tossing game. The paradigm was presented on a 21″ computer screen including instructions and pictures of the two other players the participant had been introduced before (always a woman and a man displayed as silhouettes, see Figure 1). Participants were told that all players were connected via a computer network and made their own decisions when it was their turn to toss the ball. The paradigm consisted of an inclusion block during which the participants were actively engaged in the game (mean duration 69 s). This means that the participants received the ball several times and they had to decide to pass it on to one of the other players by pressing either the right or the left arrow button on the keyboard placed in front of them. After 15 passes in total, of which the participant received the ball up to 5 times, a second block followed during which the participants were not actively involved in the game anymore. Instead, they had to passively watch the other players passing the ball to each other without receiving the ball (duration approx. 125 s). This second block was divided for further analyses into an exclusion phase followed by a habituation phase (each approx. 60 s). All participants experienced the inclusion block before the exclusion and habituation blocks. Fixing the block sequence was a deliberate choice which aimed to increase the feeling of non-belonging responses when switching from inclusion to exclusion.

### 2.4 Manipulation check

Two manipulation checks were conducted after completing the experimental paradigm. First, four statements on a 6-point-Likert-scale assessing the perceived inclusion/exclusion (e.g.. I felt as part of the group. “I felt excluded”) were presented. Second, two statements were administered assessing whether or not the participant thought he played with a real person (“I felt like playing with two other persons. “I felt like playing with a computer”) (80).

### 2.5 Physiological recording and processing

Electrodermal activity (EDA) and cardiac activity (ECG) were recorded before and during the cyberball paradigm with a bioamplifier (Mobi8-BP 12 channel amplifier, TMSi B. V., Enschede, The Netherlands) with a sampling rate of 1024 Hz and a 24-bit analog-to-digital (A/D) conversion rate. The recorded data were divided into a resting, inclusion, exclusion, and habituation^4^ phase (each approximately 60 s, see Figure 1). Portilab 2.0 software was used to acquire timing information of the different phases of the cyberball paradigm.

### 2.6 Electrodermal activity

EDA was recoded exosomatically with a direct current (DC) of 5 μV. Two Ag/AgCl electrodes were fixed to the medial phalanx of the middle- and ring-finger of the nondominant hand. Participants were asked to wash their hands with curd soap prior to electrode application.

Raw data was preprocessed in Matlab (R2020b) via the Ledalab toolbox (V3.4.9) (83, 84). EDA data was down-sampled (32 Hz), filtered (IRR Butterworth low pass filter, 4th order, 8 Hz), and smoothed using Matlab functions. Artifacts were first identified as outliers by default settings implemented in Ledalab then double-checked visually by the authors, and finally removed manually on the individual level using Ledalab’s linear interpolation function. Furthermore, a Continuous Decomposition Analysis (CDA; default settings, amplitude threshold 0.01 μS) was conducted in Ledalab to extract the mean skin conductance level (SCL), the frequency of above-threshold skin conductance responses, and the amplitude of these significant skin conductance responses separately for each cyberball phase [see (85)]. The assessment of the three different electrodermal activity measures allowed to distinguish between tonic (SCL) and phasic (frequency and amplitude of significant SCRs) aspects of an arousal response (83, 84). Each cyberball phase lasted for about 60 s, which is longer than analysis intervals usually used with SCRs. Therefore, reporting both overall SCR amplitudes as well as the number of significant SCRs was intended as a comprehensive description of individual arousal reactivity within each participant. One participant high on Fearless Dominance was not available for further analysis due to a recording error.

### 2.7 Cardiac activity (ECG)

ECG was recorded in a bipolar setting. One Ag/AgCl surface electrode was placed directly under the collarbone on the right body side, while the second Ag/AgCl electrode was placed on the left body side about 10 cm below the navel. The reference electrode was placed behind the left ear. Participants’ skin was cleansed using alcohol prior to electrode application.

Raw data was filtered and preprocessed via OpenANSLAB (V.4.0) (86) using default settings. Where necessary, the threshold criterion for detecting R-waves was adjusted in order to either reach more conservative detection levels (i.e., to reduce false positive detections in noisy data files), or more lenient detection levels (i.e., to reduce false negative detections in noisy files). Each QRS complex, respectively R-wave, was first identified by default settings in OpenANSLAB and then inspected visually on an individual level. Obviously missing or incorrectly detected R-waves were manually corrected in OpenANSLAB. Mean heart rate (HR; i.e., the number of beats per minute) was extracted for further analysis. Changes in heart rate are assumed to reflect the so-called orienting response (87), which can be described as a reflex-like response to unexpected and potentially important environmental changes.

Two participants high on Fearless Dominance were not available for HR analysis due to recording errors.

### 2.8 Statistical analyses

For statistical analyses, SPSS® v28, Jamovi 2.2.5 (88), and R 4.2.2 (89). were used. *p*-values <0.05 were interpreted as significant. p-values related to tests assessing the same hypothesis were corrected for multiple comparisons, and if available effect sizes are reported, among others to evaluate the potential clinical relevance of results.

The boxplot-method implemented in SPSS was used to identify extreme outliers on the group-level for each dependent variable (self-report, EDA, and ECG data) in each phase (resting, inclusion, exclusion, and habituation). Outliers were defined as individual mean values below Q1-IQR*3 and above Q3*IQR*3. These were only detected in EDA data and were corrected by the next minimum, respectively, next maximum value of the corresponding condition (90). Self-report data were analyzed with robust statistical models to account for violations of normality and unequal group size. Physiological data were analyzed with linear mixed models which are more robust in terms of data and sample characteristics (91).

### 2.9 Group characteristics and self-report data analysis

Sample characteristics (age, Coldheartedness, depression, social anxiety, impulsivity, anger disposition, and manipulation checks) and manipulation check questions were compared for group differences (Fearless Dominance vs. Self-Centered Impulsivity) using robust independent samples t-tests. In order to investigate the subjectively perceived effects of social exclusion, self-report data (derived from SAM, PANAS, and STAXI) was analyzed using robust mixed ANOVAs with the between-subject factor group (Fearless Dominance vs. Self-Centered Impulsivity) and the within-subject factor time point (before vs. after the cyberball paradigm). The function *bwtrim* (with the default value of 20% trimmed means) from the R package WRS2 1.1–4 (92) was used for these computations. The explanatory measure of effect size ξ (93) is reported for robust independent comparisons, while a robust version of Cohen’s d (94) is reported as effect size for dependent comparisons. Values of ξ = 0.1, 0.3, and 0.5, respectively, *d* = 0.2, 0.5, and 0.8 denote small, medium, and large effects.

EDA (SCL, frequency and amplitude of significant skin conductance responses) and HR data were analyzed with linear mixed models in Jamovi. In these analyses, group (Fearless Dominance vs. Self-Centered Impulsivity) and cyberball phase (resting, inclusion, exclusion, habituation) were modeled as fixed effects. The random effects structure included a random intercept for participant (model description: dependent variable ~1 + group + cyberball phase + group:cyberball phase + (1ǀparticipant)) No random slopes for cyberball phase were added because these models did not converge. The Satterthwaite method for approximation of degrees of freedom was used and a restricted maximum likelihood estimation was applied for fixed effects. Post-hoc tests were corrected for multiple comparisons (Holm). Semi-partial R^2^ (95) is reported as effect size, with values of 0.02, 0.13, and 0.26 denoting small, medium, and large effects (96), respectively.

In exploratory analyses, we tested whether changes in self-report and psychophysiological responses to social exclusion were associated with each other. We first computed the difference between the subjective state ratings from before to after the cyberball paradigm (post-cyberball minus pre-cyberball, denoted via “Δ” in front of the respective rating) and the difference between the exclusion and the resting phase for those psychophysiological outcomes that showed significant differences over the different cyberball phases (denoted via “Δ” in front of the respective physiological variable). With these difference scores reflecting potential changes due to social exclusion, Spearman correlations were computed per group. Per subjective state rating, corrections for multiple comparisons were implemented (2 groups x 3 psychophysiological variables; Bonferroni-corrected *p* < 0.0083).

## 3 Results

### 3.1 Group characteristics

Participants in the two groups did not differ significantly from each other concerning age (*p* = 0.311) and Coldheartedness (*p* = 0.100). In line with previous research (see (69)), participants in the Self-Centered Impulsivity group had higher scores on scales assessing depressive symptoms [Yuen’s *t* (11.8) = 4.73, *p* < 0.001, ξ = 0.90], social anxiety tendencies [Yuen’s *t* (23.5) = 5.98, *p* < 0.001, ξ = 0.89], impulsivity [Yuen’s *t* (15.9) = 3.34, *p* = 0.004, ξ = 0.69], and anger disposition [Yuen’s *t* (14.2) = 2.93, *p* = 0.011, ξ = 0.63] than participants in the Fearless Dominance group – see Supplementary Table S1. Taken together, these results indicate that the investigated participants reflect a sample that validly represents the common traits related to the two psychopathy subfactors.

### 3.2 Self-report before and after the cyberball task

Means and standard deviations are reported in Table 2.

**Table 2:** Means and standard deviation of subjective ratings.

		Fearless dominance		Self-centered impulsivity	
		(*n* = 24)		(*n* = 17)	
		*M*	*SD*	*M*	*SD*
SAM - arousal	PRE-T1	2.29	0.81	3.76	2.14
	POST-T2	2.79	1.56	3.59	1.70
SAM–dominance	PRE-T1	5.67	1.61	5.06	1.48
	POST-T2	5.63	1.47	4.88	1.65
SAM–joy	PRE-T1	7.00	0.89	6.24	1.09
	POST-T2	6.58	1.47	5.82	1.02
PANAS–pos	PRE-T1	31.63	3.81	30.41	5.42
	POST-T2	30.33	5.55	29.82	7.54
PANAS–neg	PRE-T1	11.42	1.50	14.18	4.56
	POST-T2	10.92	1.44	12.00	1.87
PANAS–affective polarity	PRE-T1	18.29	1.99	19.47	4.20
	POST-T2	17.33	2.20	17.47	2.76
STAXI–anger	PRE-T1	10.17	0.48	12.00	3.95
	POST-T2	11.29	2.07	11.53	1.55
Manipulation check items	*Being ignored*	3.17	1.20	3.53	1.28
	*Being part of a group*	2.63	1.01	2.06	1.09
	*Feelings of inclusion*	2.38	0.97	2.18	0.95
	*Feelings of exclusion*	3.25	1.03	3.35	1.17
	*Playing with individuals*	2.17	1.34	2.18	1.43
	*Playing with computer*	4.13	1.33	4.12	1.22

#### 3.2.1 Arousal

Participants in the Fearless Dominance group reported lower arousal than participants in the Self-Centered Impulsivity group [*F* (1,21.28) = 4.39, *p* = 0.048, ξ = 0.60]. Time point (*p* = 0.491) and the interaction group x time point (*p* = 0.155) were not significant.

#### 3.2.2 Dominance

The robust ANOVA did not show significant results (all *p*’s > 0.179).

#### 3.2.3 Joy

Participants in the Fearless Dominance group reported to experience higher joy than participants in the Self-Centered Impulsivity group (*F*(1,27.05) = 5.00, *p* = 0.034, ξ = 0.44). Joy ratings of all participants were higher before than after the cyberball [*F* (1,29.53) = 5.40, *p* = 0.027, ξ = 0.33]. The interaction was not significant (*p* = 0.989).

#### 3.2.4 Positive affect

The robust ANOVA did not show significant results (all *p*’s > 0.374).

#### 3.2.5 Negative affect

Participants in the Fearless Dominance group reported lower negative affect than participants in the Self-Centered Impulsivity group [*F* (1,23.46) = 6.57, *p* = 0.017, ξ = 0.49]. Time point (*p* = 0.070) and the interaction group x time point (*p* = 0.668) were not significant.

#### 3.2.6 Affective polarity

Ratings of affective polarity decreased from before to after the cyberball task [*F* (1,29.59) = 4.78, *p* = 0.037, ξ = 0.30]. Group (*p* = 0.626) and the interaction group x time point (*p* = 0.584) were not significant.

#### 3.2.7 Anger

Anger ratings increased from before to after the cyberball task [*F* (1,23.02) = 5.20, *p* = 0.032, ξ = 0.39]. Group (*p* = 0.075) and the interaction group x time point (*p* = 0.984) were not significant.

#### 3.2.8 Manipulation check

Participants in the Fearless Dominance and the Self-Centered Impulsivity group did not differ in the manipulation check questions (all *p*’s > 0.113).

In summary our results show general group-specific state differences on the one side (i.e., higher state arousal and negative affect in individuals with secondary psychopathy traits, but higher experience of joy in individuals with primary secondary traits). On the other side, task-specific changes from before to after the cyberball paradigm were observed across all participants (i.e., decrease in joy and affective polarity, increase in anger after social exclusion). No group-specific effects of social exclusion were found in self-report.

### 3.3 Electrodermal activity

#### 3.3.1 Skin conductance level

**Figure 2:**
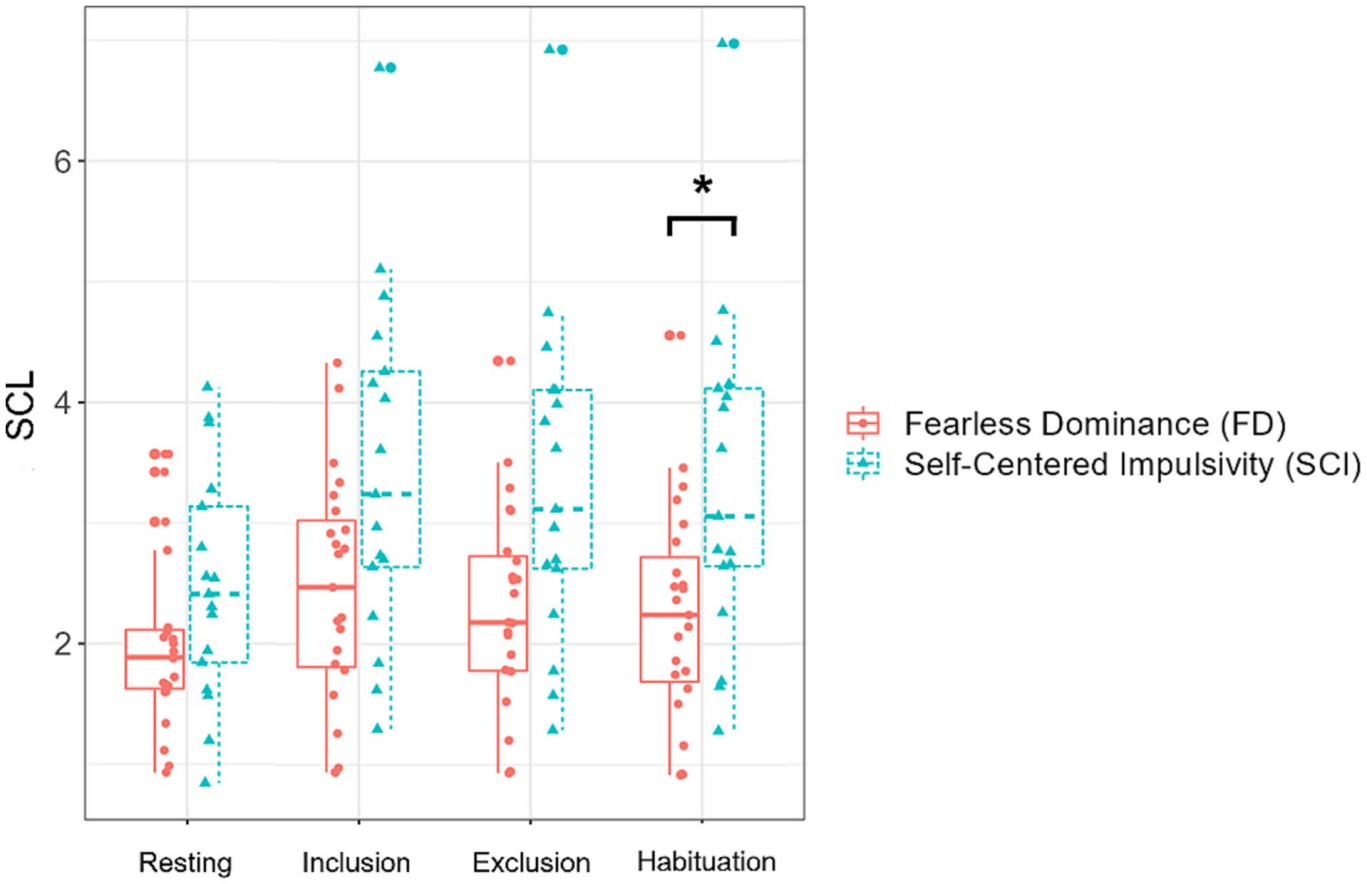
Skin conductance level values (SCL) in the two groups, separately plotted for the 4 cyberball task phases. The box plots depict the median and quartiles (whiskers demonstrating 1.5 times the interquartile range). Individual values are plotted in the respective group colors. The asterisk indicates significant group differences in *post-hoc* tests.

Skin conductance level varied significantly as a function of group [*F* (1,38) = 8.51, *p* = 0.006], cyberball phase [*F* (3,114) = 12.43, *p* < 0.001], and the interaction of group and cyberball phase [*F* (3,114) = 3.13, *p* = 0.029]. Participants in the Fearless Dominance group had lower SCL values than participants in the Self-Centered Impulsivity group [*b* = 0.91, SE = 0.31, *t* (38.0) = 2.92, *p* = 0.006, semi-partial *R*^2^ = 0.18]. Overall, SCL values increased significantly from baseline to the inclusion phase [*b* = −0.69, SE = 0.12, *t* (114.0) = −5.64, *p* < 0.001, semi-partial *R*^2^ = 0.24], and remained on a similar level when comparing inclusion and exclusion phase (*p* = 0.269) and exclusion and habituation phase (*p* = 0.927). The interaction effect seemed partly driven by a larger change in SCL from baseline to inclusion in the Self-Centered Impulsivity than the Fearless Dominance group [*b* = −0.56, SE = 0.25, *t* (114.0) = −2.29, *p* = 0.024, semi-partial *R*^2^ = 0.08]. Further disentangling the interaction effect by focusing on group comparisons at each cyberball phase showed that both groups showed comparable SCL values at baseline (*p*_holm_ > 0.999), while group differences became apparent over the course of the cyberball (inclusion: *p*_holm_ = 0.078; exclusion: *p*_holm_ = 0.056; habituation: *p*_holm_ = 0.045) – see Figure 2 and Table 3.

**Table 3:** Means and standard deviation of psychophysiological measures.

		Fearless dominance		Self-centered impulsivity	
		(*n* = 23)		(*n* = 17)	
		*M*	SD	*M*	SD
SCL	Resting	2.03	0.76	2.48	0.95
	Inclusion	2.44	0.96	3.45	1.43
	Exclusion	2.28	0.87	3.34	1.38
	Habituation	2.24	0.91	3.35	1.40
Amplitude sSCRs	Resting	0.40	0.47	0.53	0.67
	Inclusion	0.44	0.45	0.70	0.60
	Exclusion	0.40	0.41	0.86	0.95
	Habituation	0.47	0.63	1.05	1.07
Frequency sSCRs	Resting	9.09	8.64	8.71	7.09
	Inclusion	11.04	9.41	11.35	9.00
	Exclusion	11.35	10.16	13.76	11.78
	Habituation	8.78	8.23	13.53	10.73
HR	Resting	68.18	13.33	70.78	12.25
	Inclusion	68.72	11.47	72.08	14.11
	Exclusion	66.36	11.75	70.55	13.19
	Habituation	67.94	11.66	72.00	13.91

#### 3.3.2 Amplitude of sSCRs

Amplitudes of significant skin conductance responses varied significantly as a function of group [*F* (1,38.0) = 4.40, *p* = 0.043] and cyberball phase [*F* (3,114.0) = 2.87, *p* = 0.040], while the interaction was not significant (*p* = 0.146). Participants in the Fearless Dominance group had lower SCR amplitudes than participants in the Self-Centered Impulsivity group [*b* = 0.36, SE = 0.17, *t* (38.0) = 2.10, *p* = 0.043, semi-partial *R*^2^ = 0.10]. Comparisons between the cyberball phases analog to the SCL model above did not result in significant results (all *p*’s > 0.216). The observed main effect of cyberball phase was most likely driven by higher amplitudes in the habituation than the baseline phase (p_holm_ = 0.030).

#### 3.3.3 Frequency of sSCRs

No significant effects were observed (all *p*’s > 0.078).

### 3.4 Electrocardiogram

#### 3.4.1 Heart rate

**Figure 3:**
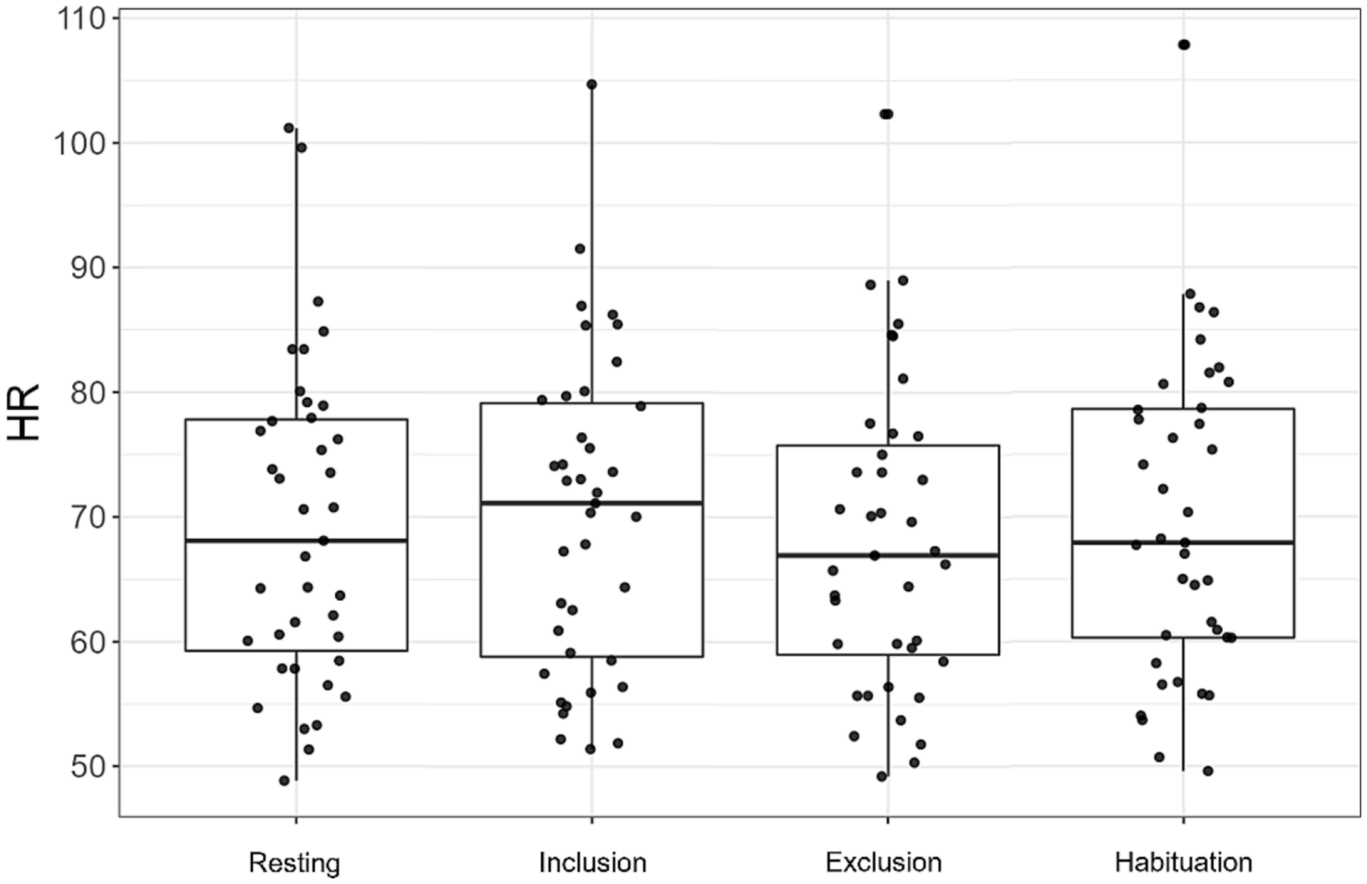
Heart rate values (HR) in all participants, separately plotted for the four cyberball task phases. The box plots depict the median and quartiles (whiskers demonstrating 1.5 times the interquartile range).

Heart rate varied significantly as a function of cyberball phase [*F* (3,111.0) = 3.42, *p* = 0.020]. While HR did not significantly change from baseline to inclusion (*p* = 0.154), it significantly decreased from inclusion to exclusion [*b* = 1.94, SE = 0.64, *t* (111.0) = 3.04, *p* = 0.003, semi-partial *R*^2^ = 0.09], and increased again from exclusion to habituation [*b* = −1.52, SE = 0.64, *t* (111.0) = −2.38, *p* = 0.019, semi-partial *R*^2^ = 0.09]. Group and the interaction between group x cyberball phase had not significant effect on HR [all *p*’s > 0.382; see Figure 3; Table 3].

### 3.5 Exploratory correlation analyses

**Figure 4:**
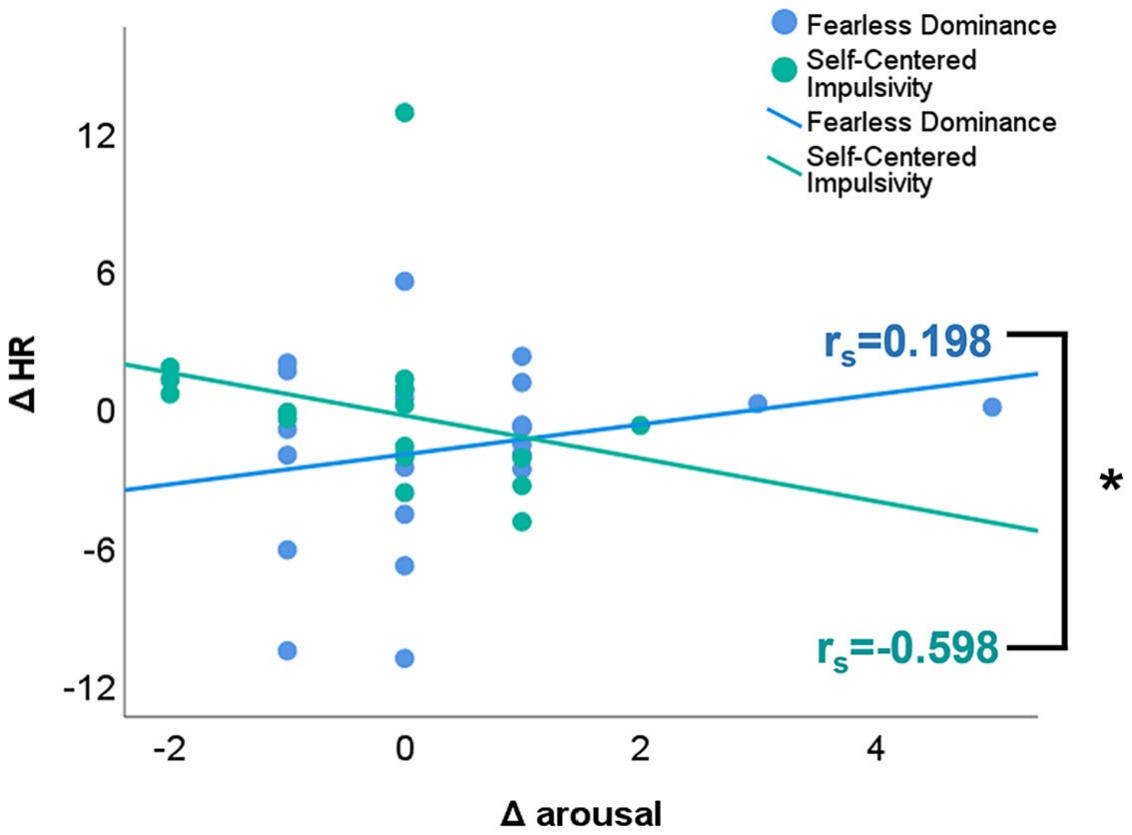
Associations between cyberball-induced changes in subjective arousal ratings and heart rate as physiological marker of arousal. “Post-cyberball minus pre-cyberball” differences in subjectively experienced arousal are plotted on the x-axis. Positive values indicate higher subjective arousal after than before the cyberball, while negative values indicate higher subjective arousal before than after the cyberball paradigm. On the y-axis, exclusion phase minus resting phase” differences are plotted for heart rate (HR). Positive values indicate higher physiological arousal during the exclusion than the resting phase, while negative values indicate higher physiological arousal in the resting than the exclusion phase. Associations are plotted for individuals high on Fearless Dominance traits in blue and for individuals high on Self-Centered Impulsivity traits in mint color. The colored solid lines depict the regression lines per group; correlation coefficients r_s_ are reported per group. The asterisk denotes a significant group difference for the association between ΔHR and Δ arousal (Steiger’s z: −2.50, *p* = 0.012).

Group-wise Spearman correlations between changes in psychophysiological variables (ΔSCL, ΔAmp_sSCR, ΔHR) and most subjective state ratings did not show strong associations (ΔDominance: *p*’s > 0.146; ΔJoy: *p*’s > 0.219; ΔPositive affect: *p*’s > 0.357; ΔNegative affect: *p*’s > 0.095; ΔAffective polarity: *p*’s > 0.056; ΔAnger: *p*’s > 0.255). In individuals high on Self-Centered Impulsivity traits, negative associations between ΔArousal and ΔSCL (r_s_ = −0.488, *p* = 0.047) and between ΔArousal and ΔHR (r_s_ = −0.598, *p* = 0.011; see Figure 4) did not pass the corrected significance level. Interestingly, however, these associations were in the opposite direction in individuals high on Fearless Dominance traits (ΔSCL: r_s_ = 0.082; ΔHR: r_s_ = 0.189, both not significant). An exploration of these diverging association patterns with Steiger’s Z test (97) to compare correlation coefficients showed that they indeed differed between the two groups for the heart rate measure (ΔArousal/ΔSCL: *z* = −1.77, *p* = 0.077, two-tailed; ΔArousal/ΔHR: *z* = −2.50, *p* = 0.012, two-tailed). Participants high on Self-Centered Impulsivity reporting higher arousal after than before the cyberball paradigm showed a reduction in physiological arousal (as indicated by HR) during exclusion compared to the resting phase. This mismatch between self-report and physiological responding was not observed in participants high on Fearless Dominance traits.

## 4 Discussion

The current study investigated the effect of social exclusion in healthy male individuals with varying psychopathy traits. More precisely, we analyzed the influence of a modified cyberball paradigm (78, 79) as a proxy for social exclusion on electrodermal and cardiac activity and on self-reported emotional states in individuals with either high Fearless Dominance (hereinafter primary psychopathy) traits or high Self-Centered Impulsivity (hereinafter secondary psychopathy) traits. It has to be noted that the characteristics of primary and secondary psychopathy cannot be equated with the characteristics of Fearless Dominance and Self-Centered Impulsivity traits and are only approximations of the corresponding factors.

Across the sample, the cyberball paradigm induced changes in self-report (e.g., increase in anger, decrease in joy and in affective polarity representing lower approach motivation and higher withdrawal after social exclusion) and physiological responding in ways that would reflect the experience of being socially excluded, with general effects comparable to previous literature [e.g., (2–6)]. These findings suggest that the current cyberball version and the experiences of our participants reflected a valid task implementation. Importantly though, individuals with high primary compared to those with high secondary psychopathy traits showed a different pattern of physiological responses when being socially excluded, which will be discussed in detail below.

As initially expected, individuals with high primary psychopathy traits reported in general significantly lower trait arousal and higher experience of joy than individuals with high secondary psychopathy traits. In contrast, individuals with high secondary psychopathy traits reported in general significantly higher negative affect than individuals with primary psychopathy traits. This is in line with previous research indicating generally low arousal levels in individuals with primary psychopathy traits (98), and the experience of predominantly negative emotions (46) in individuals with secondary psychopathy traits.

Emphasizing a divergent processing pattern of individuals high on primary and secondary psychopathy traits, physiological responses also differentiated between the two psychopathy trait groups. The amplitude of skin conductance responses was in general higher in individuals high on secondary than those high on primary psychopathy traits. Moreover, skin conductance level responses were significantly higher during the habituation phase in individuals high on secondary compared to those high on primary psychopathy traits. Furthermore, our exploratory correlation analysis showed that individuals high on secondary psychopathy traits differed significantly from individuals high on primary psychopathy traits in how physiological and self-report markers of arousal were associated with each other. While individuals with secondary psychopathy traits seemed to show a mismatch between their cognitive assessment of experienced arousal and their physiological level of arousal in response to social exclusion (i.e., self-reported higher arousal after than before social exclusion but reduction in physiological arousal as indicated by HR during exclusion compared to the resting phase), individuals high on primary psychopathy traits did not seem to be aroused at all in response to social exclusion. Taken together, these results might suggest different patterns of emotional processing and regulation during social situations in the two groups.

A possible explanation for the lack of corresponding responses of individuals with primary psychopathy traits to social exclusion in the current study can be given by characteristics associated with primary psychopathy. Generally, individuals with primary psychopathy traits were often characterized by exhibiting low physiological arousal (39) and by a tendency to show less sensitivity to social affiliation (99). Therefore, they might not feel affected by the social exclusion situation because they might simply fail to pay attention to it (41). This is in line with the proposed response modulation hypothesis of psychopathy [for a meta-analysis see (100)], in particular with the assumption of an early attentional bottleneck leading to a selective processing of only goal-directed (i.e., subjectively relevant) information while concurrently neglecting other (e.g., contextual) aspects of a situation (101). Such an early attentional focus on personally relevant information might explain why individuals with primary psychopathy traits have been assumed to not benefit from any form of treatment. However, Baskin-Sommers and colleagues (102) reported that the emotion regulation strategy of cognitive remediation was effective also in individuals with high primary psychopathy traits. Their participants benefited most from an “attention to context” training, instructing them to pay more attention to contextual cues in their environment. Linking these findings to the current study, individuals high on primary psychopathy traits might have also successfully directed their attention away from the emotionally conflicting situation. While this might be a beneficial strategy for individuals who engage too much in highly emotional situations, for individuals high on primary psychopathy traits it could be considered a maladaptive strategy, preventing them from an emotional experience in the situation at hand. Based on the findings by Baskin-Sommers and colleagues (102), future therapy programs could directly tackle this lack of emotional experience by providing instructions on what to focus on in social situations.

Individuals with high secondary psychopathy traits showed a stronger increase in physiological arousal from the resting to the inclusion phase compared to individuals with high primary psychopathy traits. Generally, an increase of arousal from the resting phase to any phase where active engagement in the experimental manipulation was required, was expected in all participants (103). The larger increase in individuals with secondary psychopathy traits as compared to those with primary ones has to be interpreted with due care. Blackburn and Lee-Evans (98) found that especially individuals with secondary psychopathy traits from a high-security psychiatric hospital report greater somatic arousal in response to anger-evoking situations. Unfortunately, to date, this association between hypo–or hyperarousal and psychopathic traits has not been sufficiently validated (40, 104). This is mainly due to methodological issues such as underpowered samples, the investigation of primarily forensic samples with interfering antisocial personality disorder, or disregarding the sub-facets of psychopathy when investigating psychopathic vs. non-psychopathic individuals (104). Therefore, a replication of our study with a larger sample would be required to draw valid conclusions on a potentially higher reactivity of individuals with secondary psychopathic traits to social situations. The assessment of physiological arousal measures, as suggested by a recent population-based study on re-offending (105), could further improve the quality of risk assessments as low autonomic arousal might serve as a predictor for reoffending.

When focusing on direct group comparisons for each cyberball phase, it seemed that participants high on secondary psychopathy traits were physiologically more reactive to the different phases of the cyberball paradigm than participants high on primary psychopathy traits. While both groups showed comparable levels of arousal (as indicated by SCL) at baseline, group differences became apparent over the course of the cyberball phases, resulting in significantly higher arousal in participants high on secondary psychopathy traits during the final habituation phase. This finding indicates a continuously high level of arousal in these individuals and is in line with previous literature assuming individuals with high psychopathic traits to show difficulties to adapt their perception or behavior in emotionally charged situations, especially when attention has already been directed toward the respective emotional stimulus (41, 42). Individuals high on secondary psychopathy traits might take a longer time until their physiological arousal levels return to resting levels. This might lead these individuals to a sustained focus on the threatening social situation and to an increase in emotional involvement. So, while individuals with primary psychopathy traits seem to not pay attention to contextual emotional cues, individuals with secondary psychopathy traits seem to show a sustained attentional focus on them. Moreover, particularly individuals high on secondary psychopathy traits are also considered to show difficulties in emotionally relevant inhibitory processes, which in turn influence their emotion regulation abilities and emotional responses (44, 45), and might lead to impulsive and aggressive behavior. These findings have been mainly reported in criminal populations, but a similar pattern might also be observable in individuals with high psychopathy traits from a community sample, as suggested by the current study.

Interestingly though, individuals with high secondary psychopathy traits seemed to show a mismatch in their cognitively perceived and physiologically experienced emotional and arousal reactions to social exclusion, as compared to individuals with high primary psychopathy traits. This pattern is somewhat reminiscent of a dissociation between emotion and cognition that is also found in traumatized individuals [e.g., (106)]. Already Porter (107) developed a theory suggesting that secondary psychopathy might rather be a distinctive dissociative disorder and not a personality disorder, defined by a disconnection between emotion and cognition influenced by early maltreatment. In line with this assumption, individuals with secondary psychopathy traits seem to develop an inability to form early secure attachments (108) due to parental rejection and abuse (109), among other experiences of interpersonal rejection. Furthermore, investigating the influence of conflicting social situations on the development of psychopathy traits, Dishion and et al. (60) evaluated effects of early aggressive behavior on peer affiliation. They found that young boys and girls with higher levels of aggressive behavior were more likely being disliked by their classmates, and therefore most probably socially excluded. Taken together, these observations could potentially partly explain the mismatch between self-report and psychophysiology observed in individuals high on secondary psychopathic traits in the current study.

Another reason might also be that physiological and subjective responses following affective stimuli or situations do not always necessarily cohere to a large extent, as debated for example by Levenson et al. (110).

Nevertheless, and in line with other studies [e.g., (99, 111), for a review on the treatment of psychopathy see (112)], our results support the necessity of implementing specific intervention strategies for individuals scoring high on psychopathy traits, in particular for those with secondary psychopathy traits. The violation of basic emotional needs in children, such as the need for secure attachments to others, can result in early maladaptive schemas leading to maladaptive coping strategies on a cognitive, emotional and behavioral level (112, 113), and contribute to the trauma-psychopathy hypothesis explaining the development of particularly secondary psychopathy traits (107, 111). The current results should be interpreted with due care. A limitation of this study pertains to the rather small sample size in an extreme group design, which results in less power to detect small to medium effects of divergent emotional experiences and processing in response to social exclusion in individuals with high psychopathic traits. However, in total 1,612 volunteers (445 men) were screened for psychopathic traits, and the recruited laboratory sample was well selected in regard to the two psychopathy factors. The number of recruited participants almost exactly represents the prevalence of individuals with psychopathic traits in the community [between 0.6% (57); and 4.5% (58)], and should therefore be considered a representative sample. Of note, the generalizability of our results is limited to a male-only sample with extremely high primary and secondary psychopathy traits in a normal population. Thus, factors such as variability in psychopathic traits or a valid control group (with, e.g., mean scores in psychopathic traits in a reference-based sample) and sex/gender are missing to draw broader conclusions. By choosing a male-only extreme group design, we aimed to enhance the comparability with forensic samples (59), which are mostly the targets of psychopathy research. Nevertheless, investigating a continuous distribution of psychopathy scores on each facet would have allowed a clearer picture of how the diverging facets individually (i.e., fearless dominance and self-centered impulsivity) and their combination relate to social exclusion. To address these limitations, future research should aim for multi-center studies that allow the collection of larger and more diverse participant samples. This may then provide a more accurate view on the underlying emotional, cognitive, and behavioral differences of, for example, socially excluded individuals with psychopathic traits. Moreover, future implementations of the cyberball paradigm could make use of virtual reality installments to further increase the validity of the artificially created social exclusion setting [such as (80)]. This could be particularly important when investigating individuals with varying psychopathy levels, who seem to be less sensitive to contextual cues.

It is noteworthy to mention that results of the exploratory association between physiological responses and self-reported state measures may be influenced by the chosen time-point of self-report assessment (after the habituation phase and not directly after the social exclusion phase). In order to minimize the influence of, for example, cognitive strategies to deal with the potentially threatening effects of social exclusion (114) it could be recommended to assess self-report directly after social exclusion. In this case, a presentation of shortened self-report assessment would be most feasible.

According to Mahmut and colleagues (59), non-criminal psychopathic individuals appear not to differ significantly from criminal psychopathic individuals in terms of psychopathic traits *per se*, but rather in their degree of expression. Hence, it can be assumed that the current findings are also applicable to criminal individuals with high psychopathy traits. Along these lines, also Boduzek and colleagues (115) argue that the prevalence of individuals with high psychopathy traits are rather comparable in student samples. In conclusion, we therefore suggest that our results may be most relevant to forensic populations with psychopathic traits and could lead to important implications for more efficient therapeutic interventions. A better understanding of how individuals with varying psychopathy traits react to social exclusion and generally to conflict-laden social situations might contribute to more suitable, need-specific therapy, and furthermore to a reduction of criminal acts and recidivism.

In terms of therapy approaches, it would be desirable to switch from a solely behavioral understanding of the psychopathy construct to a more clinical view to account for the specific cognitive and emotional dysfunctions in therapeutic settings (112). Hence, more research is needed in order to investigate the different emotion processing mechanisms and cognitive coping strategies by comparing the two psychopathy subtypes in both well-functioning community population with psychopathic traits and in matched forensic populations.

Finally, it is important to mention that our study focuses only on the two main factors of psychopathy, while more or less neglecting the third, maybe crucial, factor, namely Coldheartedness. Coldheartedness is supposed to reflect a lack of compassion, indifference against the emotions of others [e.g., lack of empathy for other’s pain (116, 117) and egoistic behavior (35)], and is therefore associated with empathic abilities crucial to social situations and interactions. These specific characteristics have caused the development of alternative conceptualization of psychopathy like the triarchic model (37) that entails Boldness (i.e., tendency for social assertiveness, dominance and emotional resilience), Meanness (i.e., lack of empathy and affiliative capacity as well as aggressive manipulation) and Disinhibition (i.e., boredom proneness, irresponsibility, and impulsivity) (118). Regarding the specific characteristics of Coldheartedness/Meanness that are not fully covered by none of the other facets, it might add pivotal information to our understanding of social behavior of individuals with psychopathic traits when integrating this third domain of psychopathy in further research on social exclusion or other social situations.

## Data availability statement

The anonymized raw data supporting the conclusions of this article will be made available by the authors, without undue reservation.

## Ethics statement

The studies involving humans were approved by University of Vienna ethics board. The studies were conducted in accordance with the local legislation and institutional requirements. The participants provided their written informed consent to participate in this study.

## Author contributions

NS: original idea, designed the study, acquired funding, performed the research, and data curation. KH-F and DP: supervision. KH-F: provided input to methods. NS and DP: data analyses, data visualization, and wrote the manuscript. NB and CL: provided comments on the manuscript. All authors contributed to the article and approved the submitted version.

## Footnotes

^1^For a discussion of factor models see also (25) PCL-R 2nd ed. Chapter 7 (pp. 77–86) or (26) for the PPI/PPI-R.

^2^Although there is some evidence demonstrating differences between the terms *social exclusion*, *social rejection* and *ostracism* [e.g., (53); for a definition of terms see (3)], they are usually used interchangeably (3). In the present manuscript these terms are used interchangeably.

^3^Invited volunteers participated in a larger research project, outside of the scope of this manuscript. Results of the other parts of the study will be reported elsewhere.

^4^It is regularly observed that, after an initial orientation response, electrodermal activity (EDA) tends to habituate (i.e., show reduced skin conductance responses) with repeatedly presented identical stimuli over time (80). The exact time frame of habituation is not well-understood and may vary, depending on, e.g., individual sensitivity to the stimuli, the intensity of the stimuli, the frequency of exposure, etc. (81). In our study, we assumed exclusion to induce cognitive and emotional processing (e.g., cognitive restructuring, switching of attention, etc.), and corresponding physiological changes, including a change in EDA during the 2 min of social exclusion. Thus, we divided the exclusion phase into two phases (each 60s) and labeled the first phase “exclusion” and the second phase “habituation.”
