# Supplementary material for: Social exclusion evokes different psychophysiological responses in individuals high on the psychopathy facets fearless dominance and self-centered impulsivity
Source: Front Psychiatry. 2024 Jan 11;14:1197595. doi: 10.3389/fpsyt.2023.1197595 (PMC10808528; doi:10.3389/fpsyt.2023.1197595)
Supplement: Supplementary file 1 [file Data_Sheet_1.docx]

**SUPPLEMENTARY MATERIALS**

**Social exclusion evokes different psychophysiological responses in individuals high on the psychopathy facets Fearless Dominance and Self-Centered Impulsivity**

Natalia A. Seeger, Natalie Brackmann, Claus Lamm, Kristina Hennig-Fast, and Daniela M. Pfabigan

Contact information: [natalia.a.seeger@gmail.com](mailto:natalia.a.seeger@gmail.com)

1. Additional information on participant recruitment of the Low-Trait psychopathy group

Also individuals with low scores (bottom 25%, based on the norm reference group) on both subscales of the Psychopathic Personality Inventory – Revised (PPI-R; [1]) were invited to the laboratory. This group was called Low-Trait group (LTG). Only 13 individuals fulfilled the criteria for the LTG group. Note that participants belonging to the LTG were scoring extremely low on both psychopathy factors. Thus, they were representing rather another extreme group (than a control group) characterized by e.g., emotional sensitivity, high fearfulness and planfulness (as opposites to high manifestations in the single PPI-R scales), more associated with neuroticism. PPI-R scores of these individuals: Fearless Dominance: M=102.92, SD=10.05; Self-Centered Impulsivity: M=118.08, SD=7.09. This is the reason why analyses including LTG individuals are presented in Supplementary Materials only.

1. Statistical analyses comparing the three experimental groups

All data, except the HR data, were analyzed with non-parametric statistical approaches because the assumption of normally distributed data was violated (assessed via Shapiro-Wilk tests).

Sample characteristics (age, coldheartedness, depression, social anxiety, impulsivity, anger disposition, and manipulation checks) were compared for group differences using Welch’s F-test. In order to investigate the subjectively perceived effects of social exclusion, self-reported state data (derived from the SAM, PANAS, and STAXI) was analyzed using Wilcoxon signed-rank test for within-group comparisons (before vs. after the cyberball paradigm), and Kruskal-Wallis test for between-group comparisons (Fearless Dominance vs. Self-Centered Impulsivity vs. Low-Trait group).

EDA data (SCL, frequency and amplitude of skin conductance responses) was analyzed using Friedman’s test for within-group comparisons (cyberball phases) and Kruskal-Wallis test for between-group comparisons (Fearless Dominance vs. Self-Centered Impulsivity vs. Low-Trait group). ECG data (HR) was analyzed using a mixed-model ANOVA with the within-subject factor cyberball phase (resting, inclusion, exclusion, habituation) and the between-subject factor psychopathy group (Fearless Dominance vs. Self-Centered Impulsivity vs. Low-Trait group). In cases of violations of sphericity, the Greenhouse-Geisser correction was applied.

1. Results of the group comparisons including the Low-Trait psychopathy group

GROUP CHARACTERISTICS

The three groups did not differ significantly from each other concerning age (p=.240) and Coldheartedness (p=.341). In line with previous research (see [62]), there was a significant group difference in depression scores (BDI-II, [2]) (F(2, 32.38)=2.036, p<.001; with descriptively highest scores in participants high on Self-Centered Impulsivity); social anxiety scores (SIAS, [3]) (F(2, 24,95)=21.070, p<.001; with descriptively lowest scores in participants high on Fearless Dominance); impulsivity scores (BIS-11, [4]) (F(2, 29.25)=4.580, p=.019; with descriptively highest scores in participants high on Self-Centered Impulsivity); and in anger disposition scores (STAXI, [5]) (F(2, 25.80)=5.661, p=.009; with descriptively highest scores in participants high on Self-Centered Impulsivity).

| **Table S1.** Means and standard deviations trait questionnaires | | | | |  |  |
| --- | --- | --- | --- | --- | --- | --- |
|  |  |  |  |  |  |  |
|  | **Fearless Dominance** | | **Self-Centered Impulsivity** | | **Low-Trait Group** | |
|  | (n=24) |  | (n=17) |  | (n=13) |  |
|  | *M* | *SD* | *M* | *SD* | *M* | *SD* |
| **Coldheartedness** | 33,58 | 5,61 | 30,76 | 6,43 | 31,85 | 5,80 |
| **BDI-II** | 3,46 | 3,16 | 15,12 | 9,16 | 7,08 | 4,17 |
| **SIAS** | 11,63 | 7,82 | 30,47 | 10,35 | 24,38 | 14,14 |
| **BIS-11** | 55,75 | 6,22 | 65,29 | 11,90 | 56,46 | 5,01 |
| **STAXI** | 68,00 | 6,05 | 76,59 | 9,09 | 69,38 | 8,55 |
|  |  |  |  |  |  |  |
| **Age** | 26,54 | 8,39 | 24,18 | 5,74 | 22,85 | 2,67 |

SELF-REPORT BEFORE AND AFTER THE CYBERBALL TASK

*Arousal*: There were no within-group differences from before to after social exclusion (all p-values > .222). Before social exclusion, there was a significant between-group difference (p=.038) which, based on post-hoc comparison, was related to participants in the Fearless Dominance group reporting significantly lower arousal than participants in the Self-Centered Impulsivity group (p=.015 / p_corr_=.045). After social exclusion, no group difference was observed (p=.097).

*Dominance*: There were no within-group differences from before to after social exclusion (all p-values > .164). Before social exclusion, no between-group difference was observed (p=.053). After social exclusion, there was a significant group difference (p=0.043). Post-hoc comparison showed that participants in the Fearless Dominance group reported significantly higher dominance than participants in the Low-Trait group (p=.017 / p_corr_=.050).

*Joy*: There were no within-group differences from before to after social exclusion (all p-values > .069). No between-group differences before (p=.086) or after (p=.082) social exclusion were observed.

*Positive affect*: Within the groups, participants of the Low-Trait group reported significantly lower positive affect following social exclusion (t(12)=3.248, p=.007). There were no group differences before (p=.545) or after (p=.242) social exclusion.

*Negative affect*: There were no within-group differences from before to after social exclusion (all p-values > .062). Before social exclusion, a significant group difference was observed (p=.025). Post-hoc comparison showed that participants in the Self-Centered Impulsivity group reported significantly higher negative affect than participants in the Fearless Dominance group (p=.011, p_corr_=.034) and initially also than participants in the Low-Trait group, but this effect was not significant after Bonferroni correction (p=.034, p_corr_=.103). After social exclusion, there was a significant group difference as well (p=.031). Post-hoc comparison showed that participants of the Fearless Dominance group reported lower negative affect than participants of the Self-Centered Impulsivity group (p=.017, p_corr_=.051) and participants of the Low-Trait group (p=.050, p_corr_=.150), while both effects were not significant after Bonferroni-correction.

*Affective polarity*: Participants of the Self-Centered Impulsivity group reported significantly lower affective polarity after social exclusion (t(16)=2.445, p=.026). There were no group differences before (p=.480) or after (p=.826) social exclusion.

*Anger*: Within the groups, participants of the Fearless Dominance group (Z=-2.751, p=.006) and the Low-Trait group (Z=-2.332, p=.020) reported significantly higher anger after compared to before social exclusion. Before social exclusion, a significant group difference was observed (p=.011). Post-hoc comparison showed that participants in the Self-Centered Impulsivity group reported significantly higher anger than participants in the Fearless Dominance group (p=.003 / corr. p_corr_=.009). There was no group difference after social exclusion (p>.576).

There was no group difference in any of the manipulation check questions (all p-values > .113).

EDA

SCL: Within the groups, participants in the Fearless Dominance group (Χ^2^(3)=11.400; p=.010) as well as participants in the Self-Centered Impulsivity group showed a significant change in SCL over time (Χ^2^(3)=13.306; p=.004). In contrast, SCL did not change significantly over time in the Low-Trait group (p=.485). No group differences were observed during the resting and inclusion phases (all p-values > .074). In contrast, during both the exclusion as well as the habituation phase, a significant group difference was observed (p=.033, respectively p=.030). Post-hoc comparisons showed that participants in the Self-Centered Impulsivity group showed significantly higher SCL than participants in the Fearless Dominance group in both phases (F(2, 49)=-12.765; p=.010 / p_corr_ = .029, respectively F(2, 49) = -12.969; p=.009 / p_corr_ = .026) – see Figure S1 and Table S2.

Amplitude of SCRs: No significant differences were observed within the groups (all p-values > .501) or between the groups in any Cyberball phase (all p-values > .378).

Frequency of SCRs: No significant differences were observed within the groups (all p-values > .139) or between the groups in any phase (all p-values > .239).


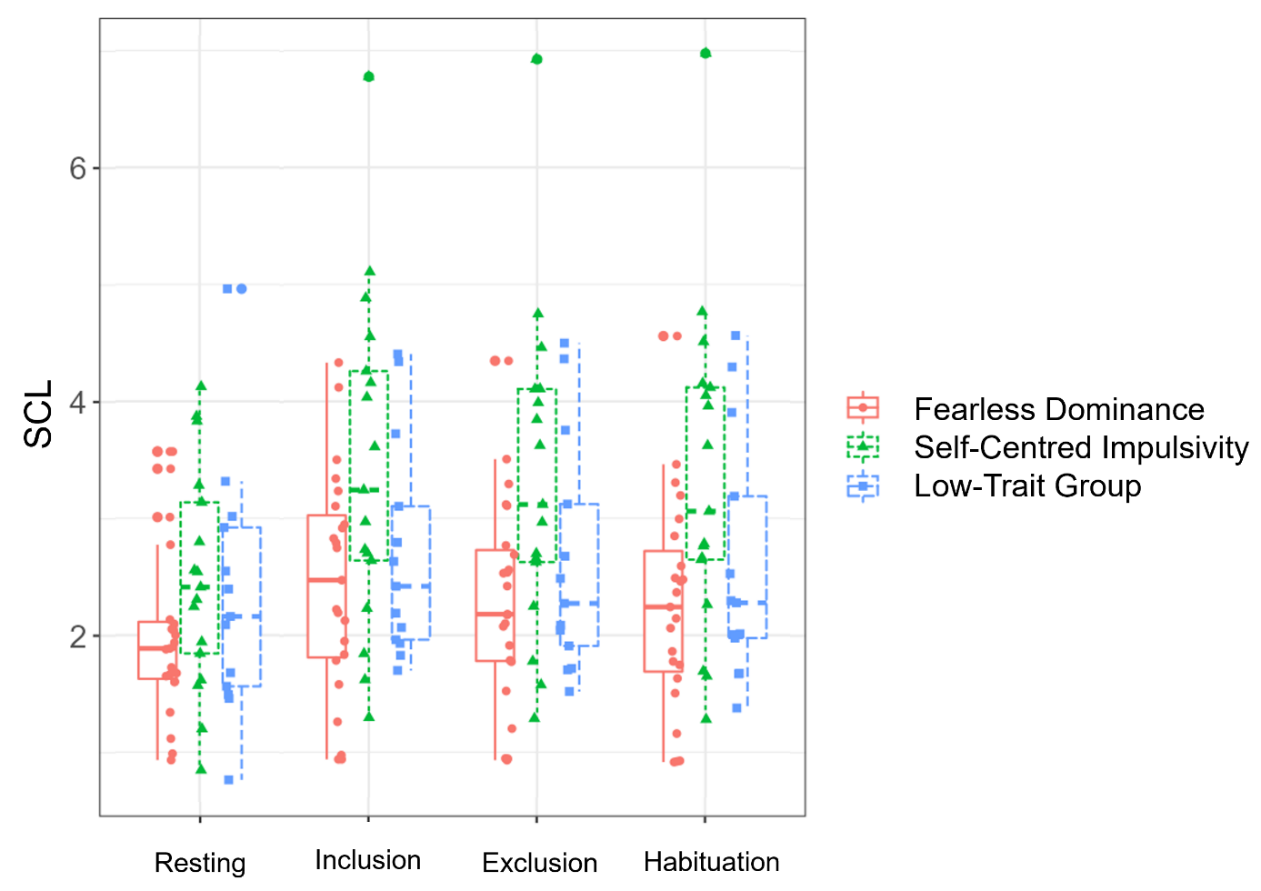


**Figure S1**. Skin conductance level values (SCL in the three groups, separately plotted for the four cyberball task phases. The box plots depict the median and quartiles (whiskers demonstrating 1.5 times the interquartile range). Individual values are plotted in the respective group colours.

ECG

*HR*: The main effect of cyberball phase was significant (F(2.39, 49)=5.520, p=.003, η_p_^2^=.101), while the main effect of psychopathy group (p=.495) and the interaction psychopathy group x cyberball phase (p=.482) were not significant. Post-hoc comparison showed a significant increase of HR from the resting phase to the inclusion phase (p=.027), a significant decrease of HR from inclusion to exclusion (p=.001), and a significant increase in HR from exclusion to habituation (p<.001), see Table S2 and Figure S2).

| **Table S2.** Means and standard deviation of psychophysiological measures | | | | | | | | | |
| --- | --- | --- | --- | --- | --- | --- | --- | --- | --- |
|  |  |  |  |  |  |  |  |  |  |
|  |  | **Fearless Dominance** | |  | **Self-Centered Impulsivity** | |  | **Low-Trait Group** | |
|  |  | (n=23) |  |  | (n=17) |  |  | (n=13) |  |
|  |  | *M* | *SD* |  | *M* | *SD* |  | *M* | *SD* |
| **SCL** | **Resting** | 2,03 | 0,76 |  | 2,48 | 0,95 |  | 2,34 | 1,07 |
|  | **Inclusion** | 2,44 | 0,96 |  | 3,45 | 1,43 |  | 2,70 | 0,93 |
|  | **Exclusion** | 2,28 | 0,87 |  | 3,34 | 1,38 |  | 2,63 | 1,01 |
|  | **Habituation** | 2,24 | 0,91 |  | 3,35 | 1,40 |  | 2,60 | 1,05 |
|  |  |  |  |  |  |  |  |  |  |
| **Amplitude nsSCRs** | **Resting** | 0,40 | 0,47 |  | 0,53 | 0,67 |  | 0,38 | 0,43 |
|  | **Inclusion** | 0,44 | 0,45 |  | 0,70 | 0,60 |  | 0,41 | 0,48 |
|  | **Exclusion** | 0,40 | 0,41 |  | 0,86 | 0,95 |  | 0,31 | 0,25 |
|  | **Habituation** | 0,47 | 0,63 |  | 1,05 | 1,07 |  | 0,43 | 0,36 |
|  |  |  |  |  |  |  |  |  |  |
| **Frequency nsSCRs** | **Resting** | 9,09 | 8,64 |  | 8,71 | 7,09 |  | 7,54 | 5,74 |
|  | **Inclusion** | 11,04 | 9,41 |  | 11,35 | 9,00 |  | 8,92 | 6,29 |
|  | **Exclusion** | 11,35 | 10,16 |  | 13,76 | 11,78 |  | 10,69 | 6,81 |
|  | **Habituation** | 8,78 | 8,23 |  | 13,53 | 10,73 |  | 10,38 | 6,83 |
|  |  |  |  |  |  |  |  |  |  |
| **HR** | **Resting** | 68,18 | 13,33 |  | 70,78 | 12,25 |  | 72,07 | 10,85 |
|  | **Inclusion** | 68,72 | 11,47 |  | 72,08 | 14,11 |  | 76,39 | 10,72 |
|  | **Exclusion** | 66,36 | 11,75 |  | 70,55 | 13,19 |  | 72,65 | 12,24 |
|  | **Habituation** | 67,94 | 11,66 |  | 72,00 | 13,91 |  | 74,39 | 12,77 |
|  |  |  |  |  |  |  |  |  |  |
| *Note*: Participant numbers for HR data are n=22 in the Fearless Dominance and n=12 in the Low-Trait Group | | | | | | | | | |


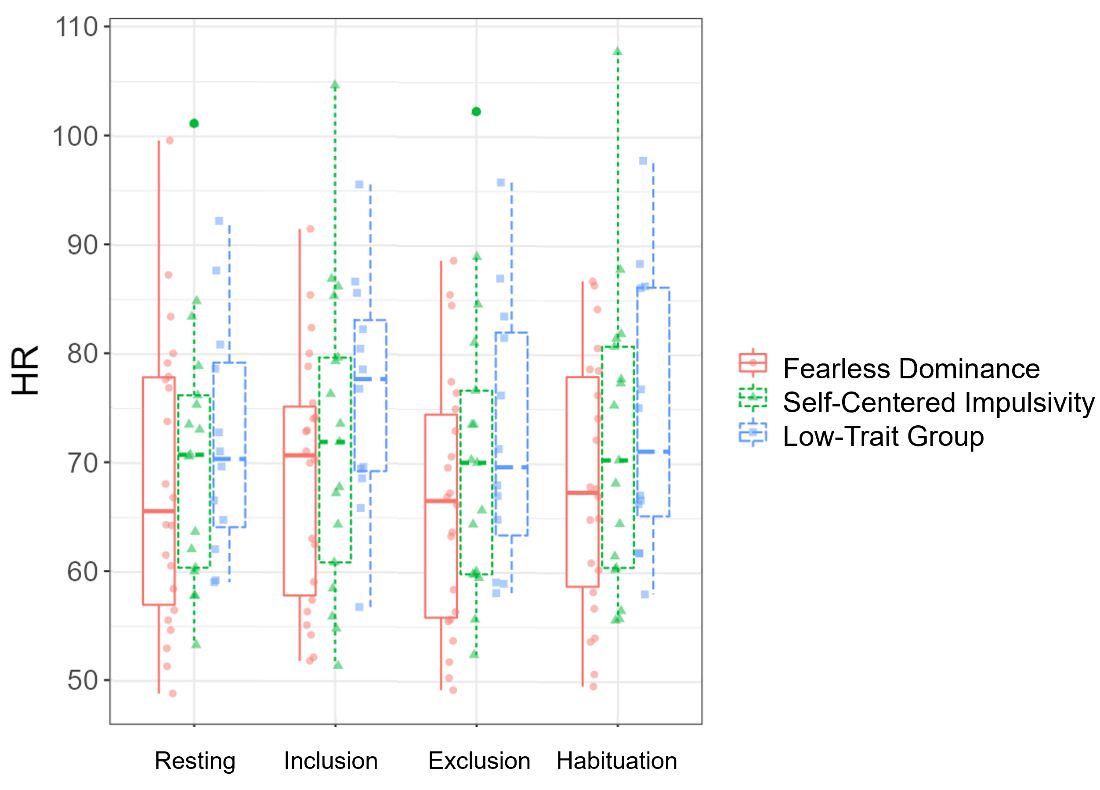


**Figure S2**. Heart rate (HR in the 3 groups, separately plotted for the four cyberball task phases. The box plots depict the median and quartiles (whiskers demonstrating 1.5 times the interquartile range). Individual values are plotted in the respective group colors.

1. Additional information regarding the manipulation check items

When asked whether they were playing with a computer or with other individuals, the current participants gave descriptively higher ratings to the first than the latter question (see Table 2 in the main document).

This might have been caused by how the two questions were framed in the German translation. Both questions were asked in a way of how much participants felt they were playing “with” - instead of “against” - a computer/a real person (e.g., “Ich hatte das Gefühl mit einem Computer zu spielen”). If participants interpreted the first question literally as “handling a computer”, the rather high ratings are not surprising as participants were in fact conducting the experiment on a computer. This could be an explanation for the high scores on that item.

Moreover, even if participants would have had the impression of playing against a computer rather than against real individuals, this should not have influenced the validity of the current findings. Previous cyberball studies have shown that being socially excluded by either real individuals or by avatars has similar effects (e.g., see [6]).

References

[1] G. W. Alpers and H. Eisenbarth, “Psychopathic Personality Inventory-Revised (German Version),” *Hogrefe: Göttingen*. 2008.

[2] A. T. Beck, R. A. Steer, and G. K. Braun, *Beck-Depressions-Inventar (BDI). Testhandbuch*, 2. Frankfurt a.M.: Hartcourt Test Service, 2009.

[3] U. Stangier, T. Heidenreich, A. Berardi, U. Golbs, and J. Hoyer, “Die Erfassung sozialer Phobie durch Social Interaction Anxiety Scale (SIAS) und die Social Phobia Scale (SPS). [Assessment of social phobia by the Social Interaction Anxiety Scale (SIAS) and the Social Phobia Scale (SPS).],” *Zeitschrift für Klin. Psychol.*, vol. 28, pp. 28–36, 1999, doi: 10.1026/0084-5345.28.1.28.

[4] J. H. Patton, M. S. Stanford, and E. S. Barratt, “Factor structure of the barratt impulsiveness scale,” *J. Clin. Psychol.*, vol. 51, no. 6, pp. 768–774, Nov. 1995, doi: https://doi.org/10.1002/1097-4679(199511)51:6<768::AID-JCLP2270510607>3.0.CO;2-1.

[5] P. Schwenkemezger, V. Hodapp, and C. D. Spielberger, *Das State-Trait-Ärgerausdrucks-Inventar STAXI: Handbuch*, 1. Auflage. Bern: Huber, 1992.

[6] O. D. Kothgassner *et al.*, “Virtual and real-life ostracism and its impact on a subsequent acute stressor,” *Physiol. Behav.*, vol. 228, p. 113205, 2021, doi: https://doi.org/10.1016/j.physbeh.2020.113205.
